# Reasoning vs. conventional large language models for BI-RADS educational questions answering: a multi-model comparative evaluation

**DOI:** 10.3389/fmed.2026.1926969

**Published:** 2026-08-28

**Authors:** Yuxia Tang, Yuting Liu, Mengxuan Liu, Hao Ni, Siqi Wang, Shouju Wang

**Affiliations:** 1 Department of Radiology, The First Affiliated Hospital with Nanjing Medical University, Nanjing, China; 2 Department of Mathematics, University College London, London, United Kingdom

**Keywords:** breast imaging reporting and data system, ChatGPT-o1, DeepSeek-R1, medical education, reasoning large language models

## Abstract

**Objective:**

To compare reasoning vs. conventional large language models (LLMs) in generating answers with guideline-aligned explanations for Breast Imaging Reporting and Data System (BI-RADS) educational questions.

**Methods:**

In this prospective study performed from February 6 to 12, 2025, 49 English-Chinese question pairs were extracted from BI-RADS Atlas Fifth Edition. Two reasoning LLMs (ChatGPT-o1, Deepseek-R1) and six conventional LLMs (Gemini2.0-Flash, Deepseek-V3, ChatGPT-4o, ChatGPT-3.5, Qwen-2.5, and WenXinYiYan-3.5) generated answers and explanations to the questions through structured prompts. Three radiologists specialized in breast imaging independently evaluated responses using a 5-point Likert scale, with reference to standard answers.

**Results:**

The reasoning LLMs significantly outperformed conventional models (median [interquartile range (IQR)]: 3.7 [2.7–4.0] vs. 2.7 [2.0–3.7], *P* < 0.001), with ChatGPT-o1 and Deepseek-R1 demonstrating peak performance. Both categories of LLMs exhibited significant score reductions in handling questions with multifaceted clinical scenarios (reasoning models: median 4.0 [2.7–4.3] vs. 2.7 [2.3–2.7], *Δ*median = −1.3, *P* < 0.001; conventional models: 2.7 [2.0–3.7] vs. 2.3 [2.0–2.7], *Δ*median = −0.4, *P* < 0.001). While question language showed no significant impact on reasoning LLMs (ChatGPT-o1 and Deepseek-R1), it affected some conventional models (ChatGPT-3.5, Deepseek-V3 and Gemini2.0-Flash). LLMs performance remained independent of question section and question type.

**Conclusions:**

Reasoning LLMs show significant potential for BI-RADS guideline explanation and education, but require specific optimization for complex clinical scenario instruction.

## 1 Introduction

Large language models (LLMs) have become pivotal tools in medicine, demonstrating utility in natural language processing, medical question answering, and clinical decision support (1–3). Their ability to encode clinical knowledge and generate contextually appropriate responses has also sparked interest in leveraging them for radiology applications, such as education, differential diagnosis, and disease classification (4–7). However, when tested on radiology board-style examination questions, conventional LLMs such as ChatGPT-3.5 exhibit adequate performance on lower-order tasks (e.g., factual recall) but demonstrate significant limitations in addressing higher-order thinking questions requiring analysis and management recommendations based on Reporting and Data Systems (RADS) (8).

The recent emergence of reasoning LLMs—exemplified by DeepSeek-R1 (released Jan. 20th, 2025) and ChatGPT-o1 (released Sep. 12th, 2024)—marks a paradigm shift in LLMs, equipping models with step-by-step logical inference capacities that allow them to handle hierarchical reasoning tasks with sequential logic (9, 10). While these reasoning models have shown promise in clinical applications like diagnostic reasoning and treatment planning (11, 12), their performance in RADS-specific higher-order thinking questions remains underexplored.

Among RADS frameworks, the Breast Imaging Reporting and Data System (BI-RADS) serves as a global standard for breast lesion characterization, management recommendations, and resident training (13–16). Despite 99% of radiology trainees in the United States and Canada receiving BI-RADS instruction during rotations, 87% report persistent challenges in guideline application and malpractice liability concerns (17, 18). Theoretically, LLMs could address these gaps through dynamic question and answer interactions that combine accurate answers with structured, context-specific explanations.

Therefore, this study aimed to evaluate and compare reasoning and conventional LLMs in generating accurate answers with structured explanations for educational questions derived from the American College of Radiology BI-RADS® Atlas.

## 2 Materials and methods

### 2.1 Questions extraction

Forty-nine English-Chinese question pairs were systematically extracted. These questions constituted the entire set of the “Frequently Asked Questions” sections of the fifth edition of the American College of Radiology BI-RADS® Atlas (both English and Chinese versions). All available questions were included to ensure representativeness, with distribution as follows: 10 question pairs from the mammography section, 11 from ultrasound, 6 from MRI, and 22 from the follow-up and outcome monitoring section. The Chinese version used was the official translation.

The educational question set was further characterized by type and complexity. Of the 49 pairs, 14 were open-ended, requiring narrative answers, while 35 were closed-ended, typically involving categorical answers. In terms of complexity, eight question pairs presented multifaceted clinical scenarios that necessitated integration of multiple situations for accurate interpretation, whereas 41 questions described straightforward scenarios with clear, singular answers. The detailed classification of each question, including its section, type, and complexity level, can be found in Supplementary Table S1. Given that all data were identified and publicly accessible within the published atlas, neither informed consent nor institutional review board approval was required for this study.

### 2.2 Response generation

**Figure 1:**
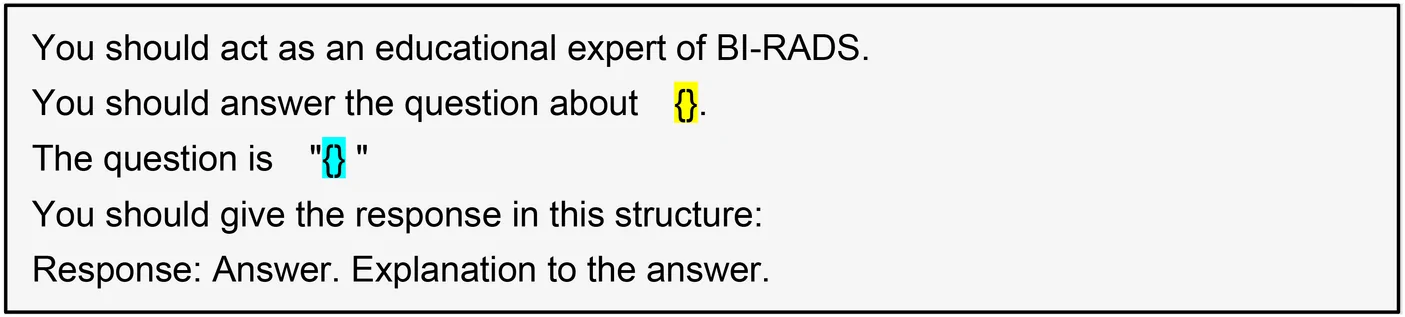
Schematic diagram of the English version of the structured prompt template. Yellow-colored placeholder denotes the question section (mammography, US, etc.), while blue-colored placeholder denotes the question stem.

A standardized prompt template was designed to elicit structured responses, incorporating role assignment, section specification, question input and formatted output requirements. See Figure 1 for template details, with colored placeholders indicating variable content. For Chinese-language questions, a translated version of the prompt template was used, maintaining identical structural elements.

Reasoning LLMs refer to models with specialized architecture or training that explicitly enhances their logical reasoning capabilities, such as native chain-of-thought reasoning. Conventional LLMs include general-purpose models without specialized reasoning optimization, which primarily rely on pattern recognition rather than structured reasoning processes. In this study, eight large language models were included. Two reasoning models, ChatGPT-o1 (OpenAI, USA) and DeepSeek-R1 (DeepSeek, China), were selected because they were the only publicly available reasoning-optimized LLMs at the time of the study. Six conventional models, ChatGPT-3.5, ChatGPT-4o, DeepSeek-V3, Gemini 2.0-Flash, Qwen-2.5, and WenXinYiYan-3.5, were chosen to represent the most advanced and widely used general-purpose LLMs from major international and Chinese platforms during the study period. Between February 6 and 12, 2025, prompts were manually entered into vendor-provided user interfaces, with each question instantiated in a fresh session to ensure response independence.

### 2.3 Response scoring

Responses were evaluated using a 5-point Likert scale: 1 indicated absent, irrelevant, or linguistically incoherent content; 2 denoted factually incorrect answers; 3 represented partially correct answers or incomplete explanations; 4 signified correct answers with comprehensive explanations; and 5 indicated correct answers accompanied by additional clinical insight or deeper analytical depth. Additionally, to ensure scoring consistency, all radiologists underwent a structured rater training session. The training materials included a detailed review of the predefined 5-point scoring rubric and a set of author-generated sample responses illustrating each score level. These sample responses were created specifically for calibration purposes and were not part of the study dataset. Three radiologists specialized in breast imaging (W.S.J.: 12-year experience, T.Y.X.: 10-year experience, W.S.Q.: 14-year experience) independently scored the response with reference to the standard answers provided by American College of Radiology BI-RADS® Atlas. All three evaluating radiologists are proficient in English and the evaluating radiologists were blinded to the LLMs’ identities.

### 2.4 Statistical analysis

Interrater reliability was assessed using Kendall's coefficient of concordance (W). Mean scores across raters were used for subsequent analyses. The Shapiro–Wilk test evaluated score distribution normality, and non-normal distributions were confirmed, justifying the exclusive adoption of nonparametric statistical tests throughout all group comparisons: the Wilcoxon rank-sum test compared reasoning versus conventional model performance, straight-forward versus complex multifaceted question performance; the Aligned Rank Transform (ART) followed by Analysis of Variance (ANOVA) was used to analyze interaction effect; the Kruskal–Wallis test assessed differences across question sections (mammography, ultrasound, MRI, follow-up and outcome monitoring); and the paired Wilcoxon test evaluated language effects (English vs. Chinese). Statistical significance was set at *P* < 0.05, with Benjamini-Hochberg correction applied for multiple comparisons. All analyses were performed using R (version 4.4.3) by W.S.J.

## 3 Results

**Figure 2:**
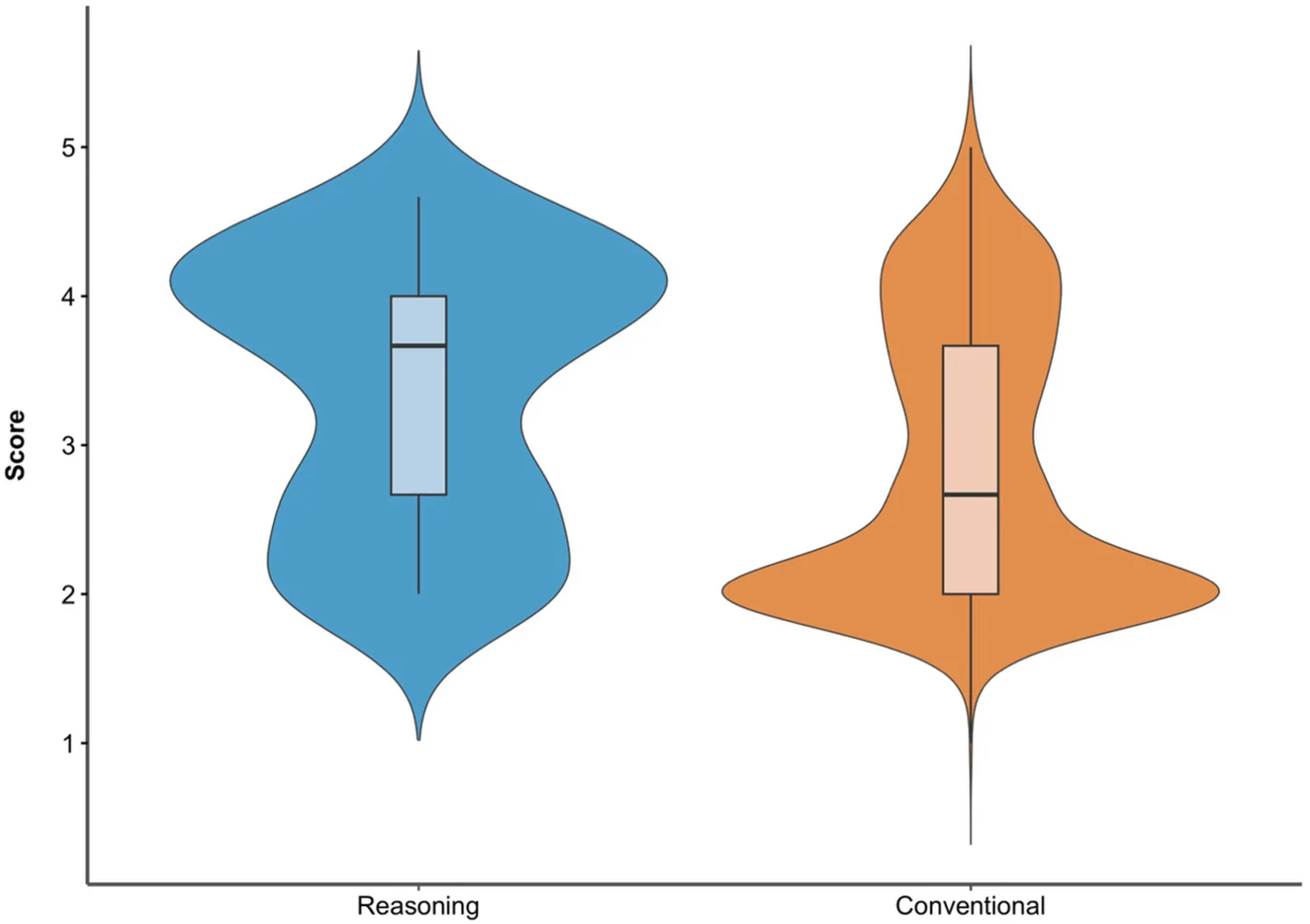
Violin plot illustrating the comparative score distributions of reasoning and conventional large language models in answering Breast Imaging Reporting and Data System educational questions. The central line represents the median score, while the box edges denote the 25th and 75th percentiles, delineating the interquartile range of the data distributions.

**Figure 3:**
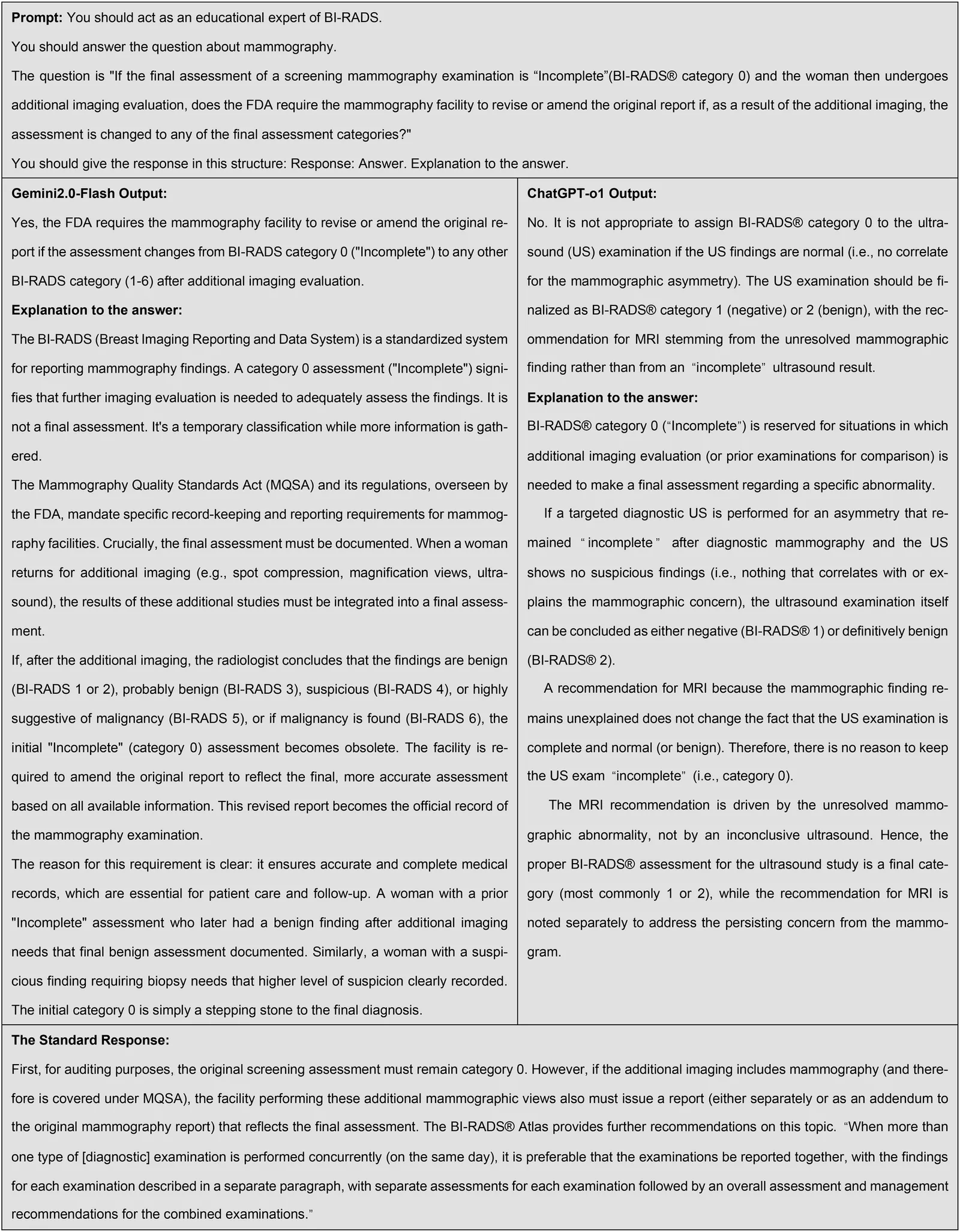
Comparison of model outputs for the same BI-RADS educational question. The standard answer from the American College of Radiology BI-RADS Atlas is shown alongside an incorrect response with factual errors from Gemini2.0-Flash (Scores: T.Y.X. 2; W.S.Q. 2; W.S.J. 2) and a correct response with comprehensive explanation from ChatGPT-o1 (Scores: T.Y.X. 4; W.S.Q. 4; W.S.J. 4), demonstrating the contrast in model performance.

High interrater reliability (Kendall's coefficient of concordance, W = 0.74) was demonstrated by the three breast radiologists, which supported the validity of subsequent score analyses. Reasoning LLMs (*n* = 2) achieved significantly higher median scores than conventional models (*n* = 6) across all BI-RADS questions [median [interquartile range, IQR]: 3.7 [2.7–4.0] vs. 2.7 [2.0–3.7], *P* < 0.001; Figures 2, 3]. The performance of individual model was listed in Table 1, with reasoning LLMs achieving the highest median scores [ChatGPT-o1: 3.7 [2.7–4.0]; DeepSeek-R1: 3.0 [2.0–4.0]].

**Table 1:** The performance of LLMs across 49 pairs of BI-RADS educational questions.

LLMs	Category	Score
ChatGPT-o1	Reasoning	3.7 [2.7, 4.0]
Deepseek-R1	Reasoning	3.0 [2.0, 4.0]
Gemini2.0-Flash	Conventional	2.5 [2.0, 4.0]
Deepseek-V3	Conventional	2.7 [2.0, 3.9]
ChatGPT-4o	Conventional	2.5 [2.0, 3.7]
ChatGPT-3.5	Conventional	2.7 [2.0, 3.7]
Qwen-2.5	Conventional	2.3 [2.0, 3.7]
WenXinYiYan-3.5	Conventional	2.3 [2.0, 3.3]

Data are presented as the median of scores, with the interquartile range (IQR) in brackets.

**Figure 4:**
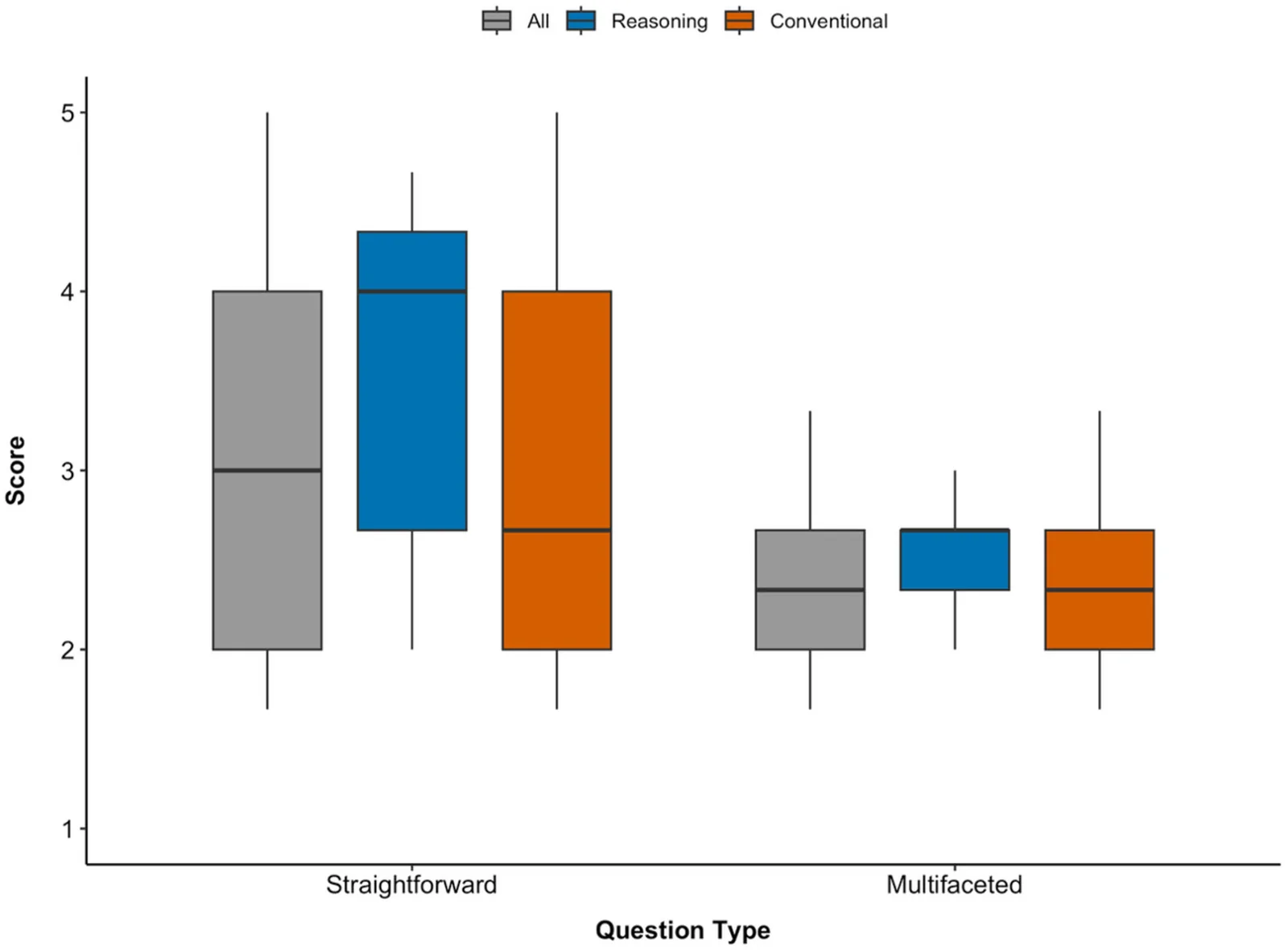
Box plot comparing the performance of all large language models (LLMs), reasoning LLMs, and conventional LLMs across two types of questions. Box components: The central line indicates the median score; box edges represent the 25th and 75th percentiles.

LLMs experienced significant score reductions when addressing complex questions involving multifaceted clinical scenarios [3.0 [2.0–4.0] vs. 2.3 [2.0–2.7], *P* < 0.001, Figure 4]. For reasoning models, the median score dropped from 4.0 [2.7–4.3] in straightforward cases to 2.7 [2.3–2.7] in multifaceted scenarios (*Δ*median = −1.3, *P* < 0.001). Conventional models saw a smaller but still significant decline, from 2.7 [2.0–4.0] to 2.3 [2.0–2.7] (*Δ*median = −0.4, *P* < 0.001). No interaction effect was detected between LLMs category and question complexity (*P* = 0.25). Detailed scoring of the eight multifaceted questions is provided in Supplementary Table S2. These questions involved scenarios requiring integration of prior imaging findings, incorporation of clinical information, and differentiation between diagnostic and screening examinations. Both ChatGPT-o1 and DeepSeek-R1 demonstrated consistently low scores across these questions.

Reasoning models showed no significant language-related performance differences (English vs. Chinese questions: ChatGPT-o1, 3.7 [2.7, 4.0] vs. 3.7 [2.7, 4.3], *P* = 0.52; Deepseek-R1, 3.0 [2.0, 4.0] vs. 3.0 [2.0, 4.0], *P* = 0.82). In contrast, several conventional models exhibited reduced scores in Chinese: ChatGPT-3.5 [English: 2.7 [2.0–3.7] vs. Chinese: 2.3 [2.0–3.3], *P* = 0.04], Deepseek-V3 [2.7 [2.0–4.0] vs. 2.3 [2.0–3.3], *P* = 0.03], and Gemini2.0-Flash [2.7 [2.0–4.0] vs. 2.3 [2.0–3.3], *P* = 0.04; Table 2]. No substantial differences were observed across question sections: mammography [3.2 (2.0–4.0)], ultrasound [2.7 (2.0–4.0)], MRI [2.3 (2.0–4.0)], and follow-up/outcome monitoring [2.7 (2.0–3.7); *P* = 0.27].

**Table 2:** Comparative analysis of language impact on LLMs performance.

LLMs	Chinese	English	*P* Value*
ChatGPT-o1	3.7 [2.7, 4.3]	3.7 [2.7, 4.0]	0.52
Deepseek-R1	3.0 [2.0, 4.0]	3.0 [2.0, 4.0]	0.82
Gemini2.0-Flash	2.3 [2.0, 3.3]	2.7 [2.0, 4.0]	**0**.**04**
Deepseek-V3	2.3 [2.0, 3.3]	2.7 [2.0, 4.0]	**0**.**03**
ChatGPT-4o	2.3 [2.0, 3.7]	2.7 [2.0, 4.0]	0.28
ChatGPT-3.5	2.3 [2.0, 3.3]	2.7 [2.0, 3.7]	**0**.**04**
Qwen-2.5	2.3 [2.0, 3.7]	2.3 [2.0, 3.7]	0.89
WenXinYiYan-3.5	2.3 [2.0, 3.3]	2.3 [2.0, 3.7]	0.53

Data are presented as the median of scores, with the interquartile range (IQR) in brackets.

* *P* Value calculated by paired Wilcoxon test and adjusted with Benjamini-Hochberg correction for multiple comparisons.

The bold values indicate that the *P* Value < 0.05.

We further quantified erroneous responses (mean rater score ≤ 2) for all LLMs (Table 3). Each model was evaluated on 49 questions per language. Reasoning models had far lower error rates: ChatGPT-o1 (English 22.4%, Chinese 12.2%) and DeepSeek-R1 (English 28.6%, Chinese 32.7%). All six conventional LLMs showed substantially higher error rates (English: 34.7–46.9%; Chinese: 44.9–49.0%). Most models produced more incorrect answers for Chinese questions; only ChatGPT-o1 maintained stable low error rates across languages.

**Table 3:** Number and proportion of factually incorrect responses (average rater score ≤ 2) for each LLM, stratified by question language.

Model	English		Chinese	
	Score ≤ 2 (*n*)	Error rate (%)	Score ≤ 2 (*n*)	Error rate (%)
ChatGPT-o1	11	22.4	6	12.2
DeepSeek-R1	14	28.6	16	32.7
DeepSeek-V3	17	34.7	22	44.9
Gemini 2.0 Flash	17	34.7	24	49.0
ChatGPT-3.5	18	36.7	24	49.0
WenXinYiYan 3.5	19	38.8	24	49.0
ChatGPT-4o	20	40.8	23	46.9
Qwen-2.5	23	46.9	22	44.9

Each model was tested on 49 distinct BI-RADS question pairs per language. Erroneous output was defined as a mean Likert score ≤ 2 across three breast imaging radiologist raters, corresponding to factually wrong or hallucinatory answers.

## 4 Discussion

Our study provides the first systematic evaluation of LLMs in interpreting American College of Radiology BI-RADS® guidelines. While LLMs have demonstrated potential in general medical question-answering, their ability to interpret radiology guidelines and potential to integrate into radiology education remains insufficiently characterized. Our results showed that the reasoning LLMs (ChatGPT-o1 and DeepSeek-R1) significantly outperformed conventional models across 49 pairs of BI-RADS educational questions. Both model categories exhibited reduced performance when addressing questions involving multifaceted clinical scenarios, though reasoning models retained higher accuracy. Language effects impacted several conventional models, but not reasoning variants, while question type and section had no significant influence. The superior performance of reasoning LLMs underscores the importance of advanced inferential capabilities in deciphering BI-RADS guidelines. Given the shortage of breast imaging radiologists (19–21), these models hold potential as interactive educational tools for residents training, particularly given their global accessibility and language-agnostic performance.

However, these models exhibited marked performance degradation when handling multifaceted clinical scenarios. These scenarios typically required context-dependent interpretation, such as integrating prior imaging, incorporating relevant clinical history, or distinguishing between screening and diagnostic examinations. Reasoning models frequently failed in these settings because they tended to apply BI-RADS rules in a context-agnostic manner. For example, when evaluating axillary lymphadenopathy, the models often assigned a BI-RADS category without considering whether the finding was unilateral or bilateral or whether infectious clinical history was present. Similarly, when assessing audit outcomes, the models frequently did not differentiate between screening and diagnostic examinations, leading to incorrect classification of positive and negative results. These error patterns closely mirror common trainee mistakes during BI-RADS learning, where failure to incorporate clinical context or examination type is a well-recognized source of misinterpretation (22, 23). Quantitative error rate analysis further confirmed this vulnerability: conventional models reached up to 49% factual error rates on Chinese BI-RADS questions, while even the top-performing ChatGPT-o1 still carried a 12.2% error risk despite its overall superiority. Identifying these specific areas of difficulty highlights the educational relevance of our findings and provides a concrete target for future LLMs optimization. Improving model performance in such multifaceted scenarios is essential for supporting accurate BI-RADS application, strengthening audit consistency, and ultimately enhancing guideline-compliant clinical practice (24).

Unlike prior studies assessing LLMs through medical examinations, our study required models to provide not only answers but also structured explanations (25–27). This dual requirement reduces the likelihood of coincidental correct answers while mirroring real-world educational needs. The models’ ability to provide guideline-referenced justifications—and occasionally generate explanations with additional clinical relevance—highlights their potential to complement traditional teaching methods.

This study has several limitations. First, the question set was constrained by the educational content available in the BI-RADS Atlas, which may limit generalizability to rare or highly complex clinical scenarios, although the included questions represent common challenges across mammography, ultrasound, and MRI. Second, the investigation was restricted to English and Chinese prompts, which precludes generalization to other languages. Third, our combined Likert scale lacks an independent conciseness metric and may penalize brief yet correct answers, and we did not test prompt engineering's potential to improve conventional LLMs. Fourth, the study focused solely on bitextual question-and-answer interactions with BI-RADS guidelines and did not evaluate model performance in image interpretation, trainee learning outcomes, or real clinical decision-making. Fifth, the small number of multifaceted questions (*n* = 8) limits the statistical power of this subgroup analysis, warranting cautious interpretation and validation in larger question sets. Finally, model performance may differ in other radiology subspecialties or under alternative question formats, such as open-ended clinical case discussions, which warrants further investigation.

## 5 Conclusions

In conclusion, reasoning LLMs demonstrate substantial potential for BI-RADS guideline interpretation and radiology education, establishing essential baseline performance metrics for their integration into training frameworks. Their ability to deliver linguistically robust explanations supports scalable training applications, though optimization for multi-context reasoning remains imperative. Future research should focus on translating these models’ interpretive accuracy into measurable improvements in trainee learning outcomes.

## Data Availability

The original contributions presented in the study are included in the article/Supplementary Material, further inquiries can be directed to the corresponding author.
