# Supplementary material for: Reasoning vs. conventional large language models for BI-RADS educational questions answering: a multi-model comparative evaluation
Source: Front Med (Lausanne). 2026 Aug 28;13:1926969. doi: 10.3389/fmed.2026.1926969 (PMC13561927; doi:10.3389/fmed.2026.1926969)
Supplement: Supplementary file 1 [file Table1.docx]

**Supplementary Table 1.** The Attributes of Questions, Including Section, Type, and Complexity Level.

| Section | Subsection | No. | Question Type | Complexity |
| --- | --- | --- | --- | --- |
| MG |  | 1 | Closed ended | Straightforward |
| MG |  | 2 | Closed ended | Straightforward |
| MG |  | 3 | Closed ended | Straightforward |
| MG |  | 4 | Closed ended | Straightforward |
| MG |  | 5 | Closed ended | Straightforward |
| MG |  | 6 | Open ended | Straightforward |
| MG |  | 7 | Closed ended | Straightforward |
| MG |  | 8 | Closed ended | Straightforward |
| MG |  | 9 | Closed ended | Straightforward |
| MG |  | 10 | Closed ended | Straightforward |
| US |  | 1 | Open ended | Straightforward |
| US |  | 2 | Closed ended | Straightforward |
| US |  | 3 | Open ended | Straightforward |
| US |  | 4 | Open ended | Straightforward |
| US |  | 5 | Closed ended | Straightforward |
| US |  | 6 | Closed ended | Straightforward |
| US |  | 7 | Closed ended | Multifaceted |
| US |  | 8 | Closed ended | Straightforward |
| US |  | 9 | Closed ended | Multifaceted |
| US |  | 10 | Open ended | Straightforward |
| US |  | 11 | Closed ended | Straightforward |
| MRI |  | 1 | Closed ended | Straightforward |
| MRI |  | 2 | Closed ended | Straightforward |
| MRI |  | 3 | Closed ended | Multifaceted |
| MRI |  | 4 | Open ended | Straightforward |
| MRI |  | 5 | Closed ended | Multifaceted |
| MRI |  | 6 | Closed ended | Straightforward |
| FOLLOW | ALL | 1 | Closed ended | Straightforward |
| FOLLOW | ALL | 2 | Open ended | Straightforward |
| FOLLOW | ALL | 3 | Closed ended | Straightforward |
| FOLLOW | ALL | 4 | Closed ended | Straightforward |
| FOLLOW | ALL | 5 | Closed ended | Straightforward |
| FOLLOW | ALL | 6 | Open ended | Straightforward |
| FOLLOW | ALL | 7 | Closed ended | Straightforward |
| FOLLOW | ALL | 8 | Closed ended | Straightforward |
| FOLLOW | MG | 1 | Closed ended | Straightforward |
| FOLLOW | MG | 2 | Closed ended | Straightforward |
| FOLLOW | MG | 3 | Open ended | Straightforward |
| FOLLOW | MG | 4 | Closed ended | Straightforward |
| FOLLOW | MG | 5 | Closed ended | Multifaceted |
| FOLLOW | US | 1 | Open ended | Straightforward |
| FOLLOW | US | 2 | Open ended | Straightforward |
| FOLLOW | US | 3 | Closed ended | Multifaceted |
| FOLLOW | US | 4 | Open ended | Straightforward |
| FOLLOW | US | 5 | Closed ended | Multifaceted |
| FOLLOW | US | 6 | Closed ended | Multifaceted |
| FOLLOW | US | 7 | Closed ended | Straightforward |
| FOLLOW | MRI | 1 | Open ended | Straightforward |
| FOLLOW | MRI | 2 | Open ended | Straightforward |

Note: A question was classified as "Multifaceted" if it met at least one of the following criteria: (a) the scenario required integration of prior imaging findings with current assessment to reach a correct BI-RADS classification; (b) the question required incorporation of relevant clinical history (e.g., presence of infection, surgical history, hormonal status) that modifies the BI-RADS interpretation; or (c) the question demanded differentiation between screening and diagnostic examination contexts. All three radiologist co-authors independently verified the classification. FOLLOW denotes the follow-up and outcome monitoring section. ALL denotes the All Breast Imaging Modalities subsection.

**Supplementary Table 2**. Detailed scoring of reasoning LLMs across BI-RADS questions involving multifaceted clinical scenarios. Data are presented as Median [Interquartile Range].

| Section | No. | Question Scenarios | ChatGPT-o1 | DeepSeek-R1 |
| --- | --- | --- | --- | --- |
| Ultrasound (US) | 7 | integrating prior imaging | 2.0  [2.0, 2.8] | 2.0  [2.0, 2.8] |
| Ultrasound (US) | 9 | distinguishing screening vs. diagnostic scenarios | 3.0  [2.2, 3.8] | 3.0  [2.2, 3.0] |
| MRI | 3 | integrating prior imaging | 2.0  [2.0, 2.0] | 2.0  [2.0, 2.0] |
| MRI | 5 | incorporating relevant clinical history | 2.0  [2.0, 2.0] | 2.0  [2.0, 2.0] |
| Follow-up (MG) | 5 | distinguishing screening vs. diagnostic examinations | 3.0  [2.2, 3.0] | 2.0  [2.0, 2.8] |
| Follow-up (US) | 3 | distinguishing screening vs. diagnostic examinations | 3.0  [2.2, 3.0] | 2.5  [2.0, 3.0] |
| Follow-up (US) | 5 | distinguishing screening vs. diagnostic examinations | 3.0  [2.2, 3.0] | 3.0  [3.0, 3.0] |
| Follow-up (US) | 6 | distinguishing screening vs. diagnostic examinations | 3.0  [2.2, 3.0] | 3.0  [3.0, 3.0] |
